# System design for inferring colony-level pollination activity through miniature bee-mounted sensors

**DOI:** 10.1038/s41598-021-82537-1

**Published:** 2021-02-19

**Authors:** Haron M. Abdel-Raziq, Daniel M. Palmer, Phoebe A. Koenig, Alyosha C. Molnar, Kirstin H. Petersen

**Affiliations:** 1 Department of Electrical and Computer Engineering, Cornell University, Ithaca, NY 14850 USA; 2 Department of Entomology, Cornell University, Ithaca, NY 14850 USA

**Keywords:** Environmental impact, Electrical and electronic engineering, Ecology

## Abstract

In digital agriculture, large-scale data acquisition and analysis can improve farm management by allowing growers to constantly monitor the state of a field. Deploying large autonomous robot teams to navigate and monitor cluttered environments, however, is difficult and costly. Here, we present methods that would allow us to leverage managed colonies of honey bees equipped with miniature flight recorders to monitor orchard pollination activity. Tracking honey bee flights can inform estimates of crop pollination, allowing growers to improve yield and resource allocation. Honey bees are adept at maneuvering complex environments and collectively pool information about nectar and pollen sources through thousands of daily flights. Additionally, colonies are present in orchards before and during bloom for many crops, as growers often rent hives to ensure successful pollination. We characterize existing Angle-Sensitive Pixels (ASPs) for use in flight recorders and calculate memory and resolution trade-offs. We further integrate ASP data into a colony foraging simulator and show how large numbers of flights refine system accuracy, using methods from robotic mapping literature. Our results indicate promising potential for such agricultural monitoring, where we leverage the superiority of social insects to sense the physical world, while providing data acquisition on par with explicitly engineered systems.

## Introduction

**Figure 1:**
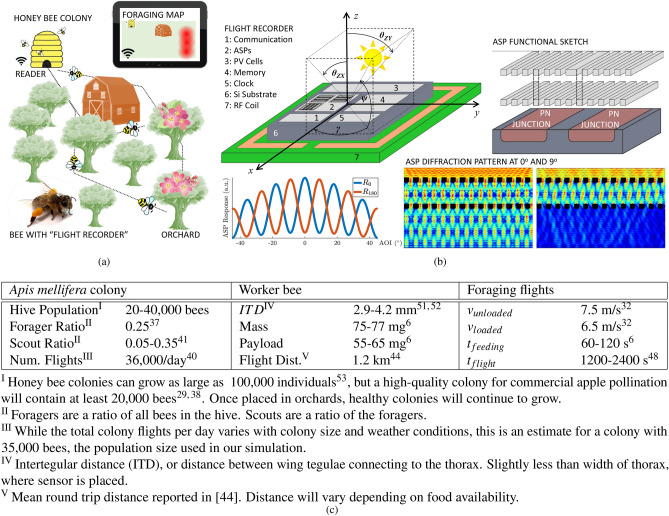
(**a**) Overview of the proposed system to monitor pollination activity through instrumented colonies of managed honey bees. (**b**) Proposed flight recorder chip layout, shown on the right, based on ASP technology, exemplified on the left. (**c**) Table summarizing relevant data pertaining to the Western honey bee.^6,29,32,37,38,40,41,44,48,51–53^

Approximately 75% of flowering plants require some degree of animal pollination, including many economically important agricultural crops^1^. Global reliance on crop pollination is expanding, yet growers world-wide suffer from increasingly unpredictable yields due to dwindling populations of wild pollinators and unsustainable losses of managed bees^2^. With some crops, including many apple varietals, having an effective pollination period of only 2 days^3^, even a slight lag in pollinator performance has immediate impacts on yield, and access to information about the state of pollination would allow growers to add pollination services when it is economical. Detecting the presence of small flowers and spurious pollination events in large fields, however, is a task far beyond the current state of the art in autonomous robots^4^. Here, we propose a novel methodology that enables use of managed colonies of *Apis mellifera*, the Western honey bee, equipped with miniature flight recorders to form inexpensive mobile sensor networks asynchronously gathering, sharing, and refining large pools of environmental information (Fig. 1a). Honey bees are the premiere agricultural pollinator, contributing more than 150B USD to the global economy annually, with an estimated 2.4 M colonies in the U.S. alone^5^. Honey bees are ideal for field monitoring: individual bees can adeptly navigate and locate flowers in complex environments, and colonies of bees can efficiently survey large areas while remaining robust to noise and partial losses^6^. First, we engineer a novel, mm-scale sensor technology based on Angle-Sensitive Pixels (ASPs)^7^ that can provide coarse trajectory tracking while meeting the strict size and weight constraints posed by the small honey bee body size and carrying capacity (Fig. 1b). Second, we combine trajectory data processing and Bayesian estimation techniques to translate this coarse data from many bee flights into refined foraging activity maps. To test our methodology, we implemented a comprehensive foraging simulator based on parameters gathered from literature on honey bees (Fig. 1c), physical orchards, and real sensor calibration.

The current trend in digital agriculture includes crop monitoring with semi-autonomous ground vehicles and drones, navigating and inspecting fields through a suite of sensors such as GPS, light detection and ranging (LIDAR), cameras, and inertial measurement units, and data analysis through multi-modal processing^4^. Although these approaches are promising for large and structured fields, robotic monitoring of small scale and temporally brief events in large, cluttered environments is difficult and prohibitively expensive. We have yet to see an autonomous system capable of estimating crop pollination state until after fruit onset. Instead, directly tapping into the abilities and knowledge of the relatively inexpensive insects that perform pollination services is tempting, and previous works have proposed both bio-mimetic^8^ and bio-hybrid solutions; the latter ranging from training^9^ and tracking^10^, to indirect control^11^ and direct control^12^. The notion of robotic and cyborg insects is promising in an engineering context, but it has not yet been practically feasible to produce long term autonomous behavior. Indirect control in social insects has been shown through automatic positive reward trainers or by mimicking existing animal communication signals^13,14^. These are promising means to influence foraging activity, however, these systems do not measure where the bees actually forage.

Tracking bees directly enables estimation of foraging locations, and indirectly, the location and pollination state of blooms. To track bees, researchers have proposed RFID tags with both passive and active transmission^15–20^, harmonic radar^21^, and LIDAR^9,21–28^. RFID technology suffers from short communication range, and harmonic radar and LIDAR require line-of-sight and expensive equipment. More recently, localization of bumble bees using RF backscatter was demonstrated in a soccer field^10^. Although smaller scale and more portable than many of the aforementioned methods, this system is restricted by the RF communication range and the volume and weight of the electronics is too large for honey bees.

In this paper, we focus on tracking honey bee foragers to estimate pollination activity in an orchard. Rather than instrumenting the landscape, we propose inexpensive flight recorders glued to the thoraxes of the bees allowing an estimated tracking distance of up to 4 km without regard for line-of-sight. Future enhancement of our flight recorder can provide even further tracking distances. These recorders periodically collect and store information on heading during flight, which is then read using backscatter communication at the hive entrance. To minimize size and weight, and thereby impact on forager behavior, we propose implementing the flight recorder in a millimeter-scale application specific integrated circuit (ASIC) complete with photovoltaic cells for power harvesting, sensors and processing circuitry, a timing clock, and memory. Central to our hypothesis is the use of ASPs to reconstruct the flight path. Through metal gratings, these pixels achieve sensitivity to incident angle of light, on the ASIC itself, without the need for any added lenses or other optical elements. With arrays of differently-oriented ASPs, we can measure the bees’ heading with respect to the sun at fixed intervals using microwatts of power^7^. We can use these data along with a motion model to reconstruct the foraging flight and feeding locations. This sampling technique is expected to suffer from quantization error and lost samples due to power dips in shaded regions, but since colonies can conduct tens of thousands of foraging flights per day and bees repeatedly visit the same bloom areas, we show how accumulated data can be processed to generate improved foraging activity maps.

Foraging activity maps may be advantageous for several reasons. Currently, pollination rates are only evident at fruit onset, when it is too late to intervene. Access to real-time foraging activity maps would permit quick and informed intervention. Honey bees are uncontrollable and may have periods of low activity or preference to other fields from where they are deployed. Knowledge about where they forage could help mitigate this loss of pollination, for example, by deploying additional biological or artificial pollinators. Previous research has shown that in apple and pear orchards, higher honey bee flower visitation rates correlate with higher fruit set, seed set, fruit-sugar content, and even increased profits^29^. Additionally, knowing when and where pollination events take place could inform better pesticide use, and potentially aid studies of disease transmission and the design of pollinator-friendly agricultural landscapes.

## Materials and methods

### Miniature flight recorders

Honey bees regularly carry a payload of 55–65 mg^6^, but a mounted flight recorder should consume only a small fraction of this allowable weight to avoid significantly affecting bee behavior. The dimensions of the recorder must also be small, as the available mounting area on the bee thorax is limited. We propose a flight recorder consisting of a $2\times 2\times 0.3\,\text {mm}^3$ ASIC mounted on a $3\times 3\times 0.4\,\text {mm}^3$ printed circuit board, which is similar in size to 3 mm diameter, 1.5 mm tall conventional bee tags (“Queen number set,” Betterbee) and is on par with previous studies using honey bee tagging methods^30^. The ASIC provides most core functionality including signal detection, memory, power harvesting, and communications circuitry, and the PCB provides a magnetic backscatter coil for near-field wireless communication. The combined weight of our chip-PCB assembly is expected to be at most 10 mg, which is a small fraction of the honey bee payload (Fig. 1c). Based on previous studies, we expect this may slightly reduce foraging trip time but not significantly impact flight characteristics, which is the focus of our system^31^. Furthermore, future iterations of our flight recorder will have smaller size and weight, minimizing the overall impact on honey bees. Power will be harvested from sunlight, which can provide intensity greater than 1 mW/mm$^2$. On-chip photovoltaics can offer power conversion efficiency on the order of 5%, supplying 50 μW of electrical power for the chip. This power budget, while low, is sufficient for the chip, since solar angle measurement and storage of data in memory are not energy-intensive operations and need only occur a few times per second. Furthermore, wireless communication for data upload is only used when the recorder is at the base station, thus allowing the base station to fully power the near-field wireless link. Additionally, IC technology is generally robust to the environmental factors likely to be encountered by honey bees. For instance, the variations in humidity level and temperature experienced by honey bees are not expected to affect chip operation. Our proposed design is a fully power-autonomous, environmentally-robust, miniature flight recorder well-suited to the task of recording honey bee activity.

The orientation of a bee during flight can be described by the *yaw* ($\gamma$), which represents the absolute heading relative to the sun, and the *angle-of-incidence (AOI)* ($\psi$), which represents the overhead angle between the sun and the sensor (Fig. 1b). To record flights, the chip uses ASPs to measure the AOI of sunlight and stores these measurements in on-chip memory. ASPs achieve AOI-sensitivity via a pair of diffraction gratings stacked over a photodiode (Fig. 1b), wherein the first grating induces a diffraction pattern that shifts laterally across the second grating as AOI is swept, thus passing a periodically-varying intensity of light to the photodiode. The stored AOI measurements can be downloaded to a base station upon return to the hive, and from these data the heading throughout the flight can be extracted. Assuming a constant speed of 6.5 m/s^32^, we can use the sequence of recorded headings to reconstruct the honey bee’s trajectory in post-processing.

The data taken by the flight recorder will be subject to measurement errors, and these errors will manifest in the reconstructed trajectory. Here, we identify and explore methods to mitigate the primary sources of error. We posit that errors will stem primarily from finite heading measurement resolution, finite sampling rate, and random fluctuations in sampling rate (*jitter*). Each of these error sources can be suppressed through careful chip design, but improvements in these performance variables can only be made at the expense of larger chip area. For instance, the heading measurement resolution will increase if more ASPs are used to measure AOI, but each pixel consumes significant silicon area and contributes additional data that must be stored in memory. Increasing the sampling rate and decreasing sampling rate jitter requires that more measurements be stored if flight time is unchanged, thus increasing the chip area required for memory. The size of the chip is thus inversely related to the severity of the expected measurement error, and trade-offs between chip size and achievable precision should be examined. A smaller sensor is feasible if trajectory reconstruction performance requirements are relaxed; more stringent requirements will necessitate a larger chip that will increase the burden on the bee. If the relationship between final uncertainty in the reconstructed position of the bee and the core sensor specifications is understood, then chip-level performance goals can be formed based on trajectory-level precision requirements.

**Figure 2:**
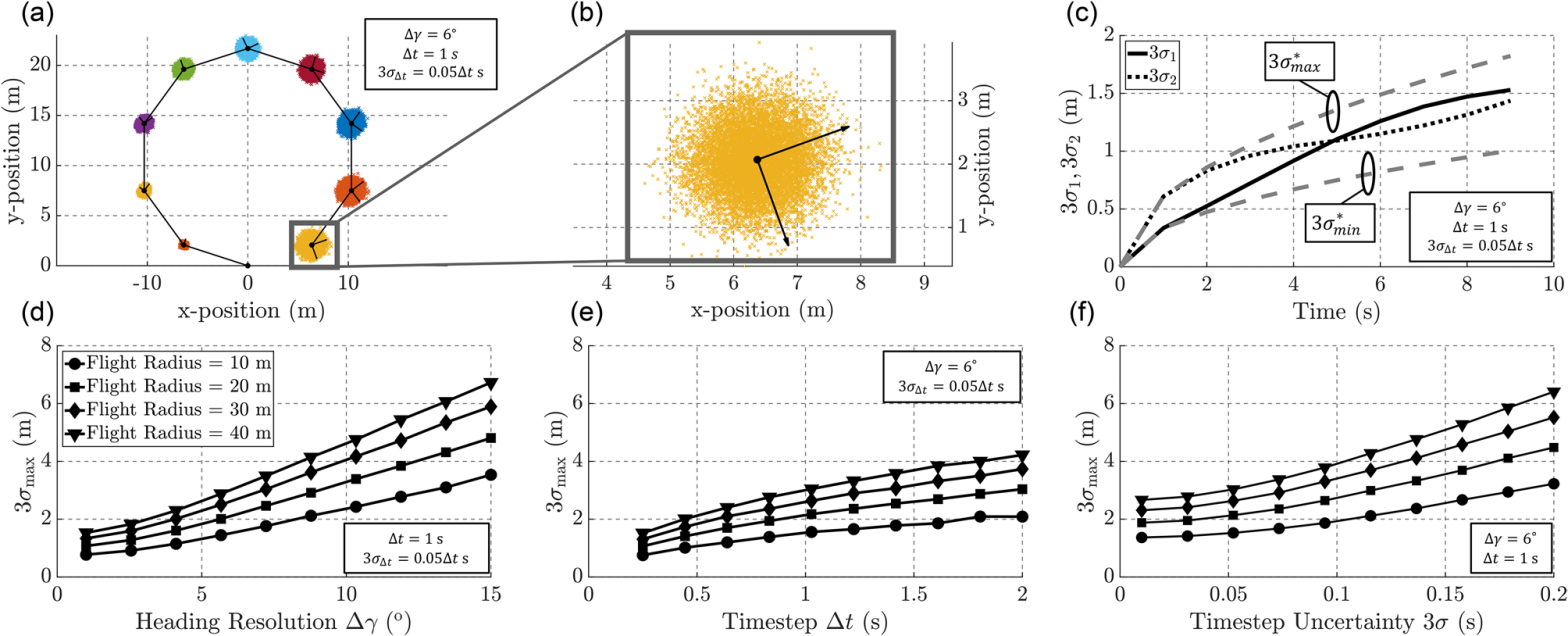
(**a**) Sensor measurement errors produce confidence regions surrounding each estimated position shown in an example circular trajectory. (**b**) Zoomed-in view of confidence region at end of flight, with principle components shown. (**c**) The two directed standard deviations grow throughout the duration of the trajectory, and are bounded above and below by the directed standard deviations computed from the case of the straight-line trajectory. (**d**–**f**) Both directional standard deviations characterizing the final error region will depend on all three of the core sensor specs $\{\Delta \gamma , \hat{\Delta t}, \sigma _{\Delta t}\}$, and the max confidence region dimension will grow if these specs are relaxed. Plots were created in MATLAB^33^.

To determine required heading resolution, sampling rate, and sampling rate jitter, we first describe the procedure to reconstruct trajectories. We define the timestep estimate $\hat{\Delta t}$ as the inverse of the sampling rate, and for known flight speed *v* the sequence of measured heading estimates $\{\hat{\gamma }_0, \hat{\gamma }_1, ..., \hat{\gamma }_{n-1}\}$ can be mapped to position estimate $\hat{\mathbf {p}}_n = [\hat{x}_n, \, \hat{y}_n]^T$ as a function of discrete time index *n* via the motion model

1 $$\begin{aligned} \hat{\mathbf {p}}_n = \sum _{i=0}^{n-1} v\, \mathbf {h}(\hat{\gamma }_i) \hat{\Delta t} \end{aligned}$$

where** h** is a unit vector pointing along the heading of the bee. Each sensor estimate of heading and timestep will be subject to errors, and by modeling these errors as additive white noise, we can evaluate a confidence region for each position estimate $\hat{\mathbf {p}}_n$ (Fig. 2a,b). Each analog heading measurement $\hat{\gamma }_i$ must be digitized for storage on-chip and will therefore suffer from quantization error, a form of rounding. We denote this error $\epsilon _{\gamma ,i}$ and model it as a uniform random variable with variance $\sigma ^2_\gamma$ on the interval $\pm \frac{\Delta \gamma }{2}$, where $\Delta \gamma$ is the heading bin width. Furthermore, the timestep estimate $\hat{\Delta t}$ will be subject to random clock jitter that can be modeled as a Gaussian random variable $\epsilon _{\Delta t,i}$ with variance $\sigma ^2_{\Delta t}$. In this model, we posit for simplicity that the random error in timing scales linearly in proportion to oscillator frequency, thus maintaining a fixed ratio of $\sigma _{\Delta t}/\hat{\Delta t}$. These measurement errors contribute random error to position estimate ${\hat{\mathbf {p}}}_n$, and thus each position estimate should be viewed as a random variable $\mathbf {p}_n$. The confidence region surrounding ${\hat{\mathbf {p}}}_n$ depends on the covariance matrix of ${\mathbf {p}_n}$, and we evaluate these terms by first using the small-angle approximation to linearize the motion model with respect to $\epsilon _{\gamma ,i}$:

2 $$\begin{aligned} \mathbf {p}_n = \sum _{i=0}^{n-1} v \, \mathbf {h}(\hat{\gamma }_i + \epsilon _{\gamma ,i}) (\hat{\Delta t}+\epsilon _{\Delta t, i}) &\approx \sum _{i=0}^{n-1} v \, \left( \mathbf {h}(\hat{\gamma }_i) + \mathbf {h}^\perp (\hat{\gamma }_i)\epsilon _{\gamma ,i}\right) (\hat{\Delta t}+\epsilon _{\Delta t, i}) = \sum _{i=0}^{n-1} v \, \mathbf {h}(\hat{\gamma }_i)(\hat{\Delta t}+\epsilon _{\Delta t,i}) + \sum _{i=0}^{n-1} v \, \mathbf {h}^\perp (\hat{\gamma }_i)\epsilon _{\gamma ,i} (\hat{\Delta t} + \epsilon _{\Delta t, i}) \end{aligned}$$

This approximation is valid if $\epsilon _{\gamma ,i}$ is kept small, which can be guaranteed by keeping heading bin width $\Delta \gamma$ small. The covariance matrix of $\mathbf {p}_n$ is then given by

3 $$\begin{aligned} \mathrm {Cov}(\mathbf {p}_n, \mathbf {p}_n) = \varvec{ \Sigma }_n \approx \sum _{i=0}^{n-1} R(\hat{{\gamma }}_{i}) \begin{bmatrix} v^2\sigma ^2_{\Delta t} &{} 0 \\ 0 &{} v^2\sigma ^2_{\gamma }(\sigma _{\Delta t}^2 + (\hat{\Delta t})^2) \end{bmatrix} {R^T({\hat{\gamma }}_i)} \end{aligned}$$

where *R* is the standard 2 × 2 rotation matrix (Appendix: Derivation of Trajectory Precision Equation). By the Central Limit Theorem, after a sufficient number of timesteps $\mathbf {p}_n$ will become Gaussian distributed. Thus, the confidence region will be an ellipse with major and minor axes spanned by the eigenvectors $\{\mathbf {v}_1, \mathbf {v}_2\}$ of ${\varvec{\Sigma }}_n$. The covariances of the confidence region along each of these axes are given by the eigenvalues $\{\lambda _1, \lambda _2\}$ of $\varvec{\Sigma }_n$, and directed standard deviations can then be defined as $\{\sigma _1,\sigma _2\}$. A 99% confidence region for position estimate ${\mathbf {\hat{p}}}_{n}$ is given by an ellipse with major and minor axes lengths $\{3\sigma _1\mathbf {v}_1, 3\sigma _2\mathbf {v}_2\}$. We therefore conclude that $3\sigma _1$ and $3\sigma _2$ are critical values defining achievable trajectory reconstruction precision.

These $3\sigma$-bounds can be computed for any measured sequence of headings, but general upper and lower bounds for $3\sigma _1$ and $3\sigma _2$ across all possible trajectories can be derived from the $3\sigma _1$ and $3\sigma _2$ given by the case in which the bee flies in a straight line. In the straight-line case, the eigenvalues of $\varvec{\Sigma }_n$ are given by *n* times the diagonal entries of the diagonal matrix in Eq. (3). The directed $3\sigma$-bounds are then

4 $$\begin{aligned} 3\sigma _{1,n}^*= & {} 3\sqrt{n} v \sigma _{\Delta t} \end{aligned}$$

5 $$\begin{aligned} 3\sigma _{2,n}^*= & {} 3\sqrt{n} v \sigma _{\gamma }\sqrt{\sigma _{\Delta t}^2 + (\hat{\Delta t})^2} \end{aligned}$$

For some values of $\{\sigma _{\gamma }, \hat{\Delta t}, \sigma _{\Delta t}\}$, $3\sigma _{1,n}^*$ will be larger than $3\sigma _{2,n}^*$; for others, the converse will be true. These equations provide simple bounds on achievable reconstruction precision that are valid for any trajectory and can be computed from sensor characteristics. Since heading error $\epsilon _{\gamma _i}$ is uniformly distributed, heading variance $\sigma ^2_\gamma$ is defined by heading bin width $\Delta \gamma$, and thus the reconstruction precision is defined by a core suite of sensor specifications: $\{\Delta \gamma , \hat{\Delta t}, \sigma _{\Delta t}\}$. An illustration of the trajectory reconstruction process, along with confidence regions, is shown in Fig. 2, as well as the relationship between reconstruction precision and each of the core chip specs.

**Figure 3:**
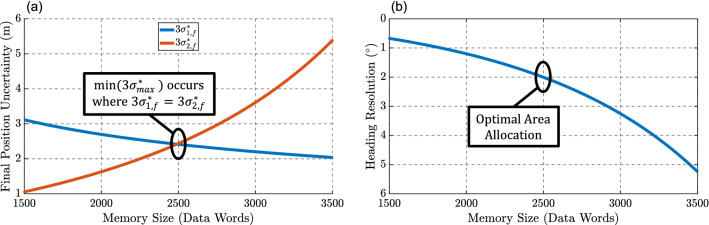
(**a**) Area usage is optimized where $3\sigma ^*_{1,f}$ and $3\sigma ^*_{2,f}$ cross, as this design point co-minimizes the two directional standard deviations characterizing trajectory precision. (**b**) This design point specifies the heading resolution and number of data words in memory that minimize trajectory uncertainty given a fixed sensor area constraint. Plots were created in MATLAB^33^.

For a maximum specified chip sensing area, trajectory precision should be optimized through balanced allocation of area to solar AOI detection and to memory (Fig. 3). We evaluate the optimal area allocation by defining the standard deviation upper bound $3\sigma ^*_{max} = \max (3\sigma _{1,f}^*, 3\sigma _{2,f}^*)$, where $3\sigma _{1,f}^*$ and $3\sigma _{2,f}^*$ are the directed $3\sigma$-values computed from a straight-line trajectory that is long enough to completely fill the memory. If more area is spent on pixels for AOI detection, heading resolution can be increased, thus causing $\sigma _\gamma$ to be reduced and correspondingly lowering $3\sigma ^*_{2,f}$. Conversely, if more area is spent on memory, measurements can be taken more frequently, and timestep and clock jitter can be reduced, thus reducing $\hat{\Delta t}$ and $\sigma _{\Delta t}$. This will cause $3\sigma ^*_{1,f}$ to decrease, but may cause an increase in $3\sigma ^*_{2,f}$ since less area is now available for heading sensors. As shown in Fig. 3, the upper bound $3\sigma ^*_{max}$ minimizes when the two counteracting variables $3\sigma ^*_{1,f}$ and $3\sigma ^*_{2,f}$ are equal, and this intersection point prescribes the optimal allocation of sensor area. Our proposed flight recorder features a 4 $\mathrm {mm}^2$ chip, which can offer sensing area of approximately 3 $\mathrm {mm}^2$. When this area is allocated optimally, the heading resolution is $2^\circ$ and timestep is approximately 240 ms at timestep jitter of $3\sigma _{\Delta t}/\Delta t = 0.03$. With these specifications, the maximum recordable trajectory length is approximately 4 km, with $3\sigma$ uncertainty of $\pm 2.4$ m.

### Sensor calibration and noise modeling

**Figure 4:**
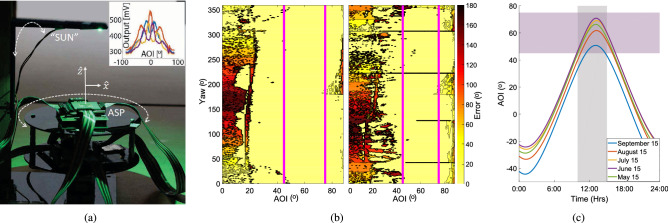
(**a**) Calibration apparatus with inset example of a measured ASP quadrature response. (**b**) ASP array repeatability over a single sensor (left) and multiple sensors (right). The magenta lines denote the expected operating region. (**c**) Expected system operating region (45°–75°) shown in magenta given the peak foraging hours (10 a.m.–4 p.m.) shown in grey and the AOI solar light during the Summer in Ithaca, NY. Plots in (**a**-inset), (**b**) and (**c**) were created in MATLAB^33^.

We next examined the output from existing ASP array sensors in order to create a model for future flight recorders. These particular sensors have 96 pixels, or 24 sets of 4 oriented in 90° angles. Specifically, we designed a calibration apparatus that consists of a platform holding the ASP array driven by a custom microcontroller PCB and an arm with a light emitting diode (LED) to imitate the sun. The platform rotates to mimic a change in yaw, while the arm rotates to mimic different AOI solar light at different times of the day (Fig. 4a). Using this apparatus, we measured light input in 0.9° increments across the entire hemisphere and record the ASP array response (Fig. 4a inset) for a total resolution of 40,000 measurements in a single sweep with 200 AOI angles and 200 yaw angles. Measurements sampled by the microcontroller were transmitted to a desktop computer for logging and processing via a custom MATLAB^33^ script. Each ASP array response consists of a 48-bit sequence. To interpret the output, we created a lookup table from the unique 48-bit sequence that is stored for each yaw-AOI angle pair. Future data was then compared to these stored sequences in parallel using an XOR operation and the pair with the least difference in bit values was returned.

We characterized sensor repeatability by repeating a sweep three times with a single ASP array and, similarly, characterized precision by comparing sweeps from two additional ASP arrays. A sample curve from the calibration sweep seen in the inset in Fig. 4a shows the characteristic angle dependence. The sensor exhibits poor response uniqueness when the sun approaches zenith and when it nears the horizon; upwards of 100$^\circ$ error in yaw near 90$^\circ$ AOI (zenith) and up to 180$^\circ$ error in yaw at 0°–25° AOI (horizon) (Fig. 4b). The former occurs because the position of a light source directly overhead is ambiguous to the sensor across yaw and consequently, indeterminate. We find that the sensor simply does not operate well in the latter region where light is arriving nearly parallel to the surface of the chip. Furthermore, as is expected, the difference in response is generally greater when comparing different sensors. The horizontal dark bars in the right graph in Fig. 4b are examples of this increased error. We expect our system to operate under favorable foraging conditions. Based on previously published data, we estimate this operating region to be May through September at an example location of the authors’ hometown of Ithaca, New York, USA, with the most active foraging hours being from 10 a.m. to 4 p.m.^34^. During this time, the AOI spans $45^\circ$ to $75^\circ$ (Fig. 4c). Within this region, we see a significantly reduced same-chip error in yaw with a mean and standard deviation of $1.52^\circ \pm 1.23^\circ$. We compensate for the remaining error within the operating region as discussed in the following sections.

In order to realistically simulate sensor output, we create a lookup table with an error model for each individual AOI value. Similar to our theoretical model, we fit a normal distribution to error in the yaw angle measured at each AOI. We use this error model to inform reconstruction of recorded bee paths as described in the following sections. For this work, we assume that we have access to calibration data for each particular sensor, however, given the low discrepancy between sensors (Fig. 4b right), we believe that it is possible to avoid individual calibration with more sophisticated data processing. We leave this aspect for future work.

### Honey bee foraging simulation

To properly develop our methodology for using instrumented bees to monitor the state of pollination and bloom, we designed a colony foraging simulator with an example apple orchard. Central to our approach is an understanding of the behavior and environmental conditions surrounding honey bee foraging, summarized in Fig. 1c. The following subsections detail orchard, honey bee motion, and colony foraging models.

#### Orchard model

**Figure 5:**
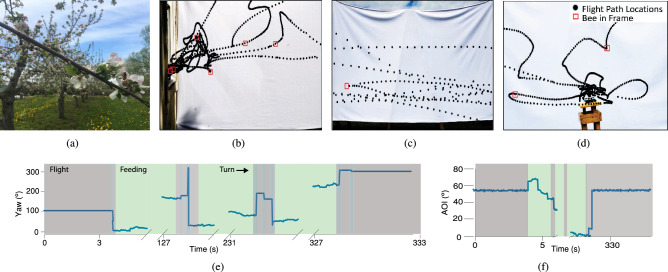
(**a**) Photo of a honey bee in a conventional apple orchard. (**b**–**d**) Setup to showcase different types of honey bee flights. Still images from videos recorded at the hive entrance, in between, and at the feeder station, with tracked paths overlaid. (**e**–**f**) Recorded heading over the course of a simulated foraging flight and feeding event. Straight line flights are marked in grey, turns in blue, and feeding events in green. Image overlays in (**b**), (**c**) and (**d**) were created in MATLAB^33^. Plots in (**e**) and (**f**) were created in Python 3.7.

We modelled the orchard based on common characteristics seen in real orchards (Fig. 5a) as well as those reported by the University of Vermont Cooperative Extension for Growing Fruit Trees^35^. Specifically, these include a tree trunk radius of 0.15 m, separation between individual trees in a given planted row as 2.4 m, and separation between rows as 5 m. To make the model realistic to a variety of orchards, we add randomness to the trunk radius (0.15–0.30 m) and to the tree locations (up to 0.5 m in any direction). We further use a 60 × 60 m^2^ area with 200 trees, as is representative of the common grower practice utilizing a single colony per acre^36^. We account for the fact that trees can be in different stages of bloom by assigning each a randomly generated quality factor between 1 and 10; this quality factor affects the number of feeding events in a flight.

#### Colony foraging model

A high-quality honey bee colony for commercial apple pollination contains a laying queen, developing brood, and 20,000–40,000 worker bees, of which approximately 25% are “foragers”, or those that leave the hive to collect pollen, nectar, resin, and water^29,37,38^. Since resin and water foragers are a small proportion of the forager workforce, we expect our flight dataset to be largely from bees that are visiting flowers^39^. For this work, we assume favorable foraging conditions as previously described and a colony size of 35,000, 25% of which are foragers for a total of 8750 foragers conducting ~ 36,000 flights per day (an average of 4 flights per forager)^37,40^. In our model, a forager can perform either a learning-, return-, or scout flight. Note that we exclude orientation flights, which are conducted by new foragers, under the assumption that these can be easily classified given their tortuous nature^23^. A learning flight is when a bee orients to a feeding site it has not previously visited after learning the bearing and distance from one of its sisters in the hive^41,42^. A return flight occurs when a bee orients to a feeding site it has previously visited, and can be thought of as an optimized version of the learning flight in terms of distance flown^43^. A scout flight occurs when a forager leaves the hive to search independently for new feeding sites. In our model, we make the assumption based on published behavioral research that 20% of foragers are acting as scout foragers and the remaining 80% perform an initial learning flight followed by return flights to the same source^41–43^.

We incorporate that return flights will frequent the same feeding sites and that neighboring trees are likely to bloom together by randomly assigning initial goal locations (trees that bees advertise in the colony as high quality food sources) to 5 neighboring trees. Bees will randomly choose between these 5, then continue feeding on neighboring trees until they have visited trees with quality factors accumulating to at least 10 before returning to the hive. Scout foragers randomly visit trees in the orchard. Realistically, not all bees will be tagged and some tags will be lost. Here, we consider a conservative estimate that at least 430 or 5% of all foragers will be tagged, leading us to 1750 recorded flights per day, and use accumulated data to overcome the loss of tagged bees, which we expect to occur as a result of predation, senescence, stress, and other factors. Note that honey bees have a pronounced division of labor associated with worker age^41^, making it easy to tag a cohort and wait for them to become foragers, or to identify foragers and tag them specifically. Tagging 430 bees would take our honey bee technician approximately a day; speeding up this process is an area of future investigation.

#### Honey bee motion model

To better illustrate the characteristics of foraging flights, we recorded activity between a queenright colony with about 10,000 workers and a nearby feeder station (Fig. 5b–d). Three distinct phases of the bee flights were recorded: an initial orientation flight upon leaving the hive, flights between the hive and the feeder, and search flights near the feeder. Flights near the entrance and the feeder were characterized by rapid turning, whereas flights in between the hive and the feeder were nearly straight “bee lines”. While at the feeder, bees crawl around at a significantly reduced velocity.

We use this study to inform our foraging simulation. We assume generally straight paths in obstacle-free environments, slow turns when avoiding obstacles, and rapid turns in AOI and yaw when nearing and crawling on a food source. We further base our flight model on the following assumptions, summarized in Fig. 1c. (1) We assume the starting location is well known since the flight path will always originate and terminate at the hive entrance. (2) Based on past studies and the fact that our simulation takes place in a dense apple orchard, we assume most flights will be within the 4 km range of our flight recorder^44–46^. When leaving the hive, bees will fly an average of ~ 7.5 m/s, but once loaded with nectar, flight speed is reduced to ~ 6.5 m/s^32^. Here, we assume a constant velocity of ~ 6.5 m/s. (3) Based on prior honey bee tracking studies^23,47^, we represent flight in only two dimensions. The apple trees in the orchards we model are not tall and bees will therefore experience much greater motion in the horizontal plane than the vertical. Regardless of whether a bee in reality will fly over or under the canopy, we can model this issue in two dimensions. Turns around tree trunks represent the largest source of error for flight reconstruction, therefore by modeling flight under the canopy, we model the “worst case” scenario. (4) We estimate that the AOI of sunlight with respect to the orchard will remain within a quantifiable margin throughout the duration of the simulated flights, as bees have been found to spend an average of 20–45 min on foraging flights^48^. (5) We model the yaw of a bee as constant during bee-line flights, i.e. given no nearby obstacles. When the bee changes its heading to circumvent obstacles, this causes a change in yaw. (6) Once implemented on bees, we expect to be able to add sensors near the hive which would help us acquire current temperature and weather patterns as well as other dynamic factors specific to a particular environment for calibrating our model.

To simulate scouting, learning, and return foraging flights, we combine the honey bee motion model previously described, with the Bug2 algorithm and grid-based path planning^49^. Grid based path planning uses a discrete grid of points over which an agent searches to find obstacle-free path segments. We compute scout and learning flights as follows. Using the Bug2 algorithm, honey bee paths are generated by first assuming direct flight along a known heading from the hive. Once an obstacle is encountered, the bee searches for a path around it, until it can once more move unhindered toward the goal. The process is repeated until all goals are reached and the bee has returned to the hive. Since no two paths are identical in nature, we plan obstacle navigation with a randomly generated grid. Return flights are found by forming a graph of all the points visited during a learning flight and using a Dijkstra’s search algorithm^49^ to find the shortest path through these points from the hive to the goal. Once paths are generated, we compute the sequence of headings given the 240 ms sensor sampling frequency reported earlier. An example flight is shown in Fig. 5e,f. These headings are then discretized based on the ASP calibration data discussed earlier, and noise is added given the error shown in Fig. 4c left.

When bees land at a feeding site, they tend to crawl on and among flowers to gather nectar and pollen. We simulate this by generating random motion centered around the feeding site. The average feeding time was reported to be 1–2 min per feeding site^6^. To make the simulation more realistic, we randomly generate a feeding time between 60 and 120 s for foragers, and between 20 and 130 s for scouts. We furthermore assume that the AOI of sunlight changes as the honey bee tilts up and down while crawling on flowers.

### Path reconstruction and generation of foraging activity maps

**Figure 6:**
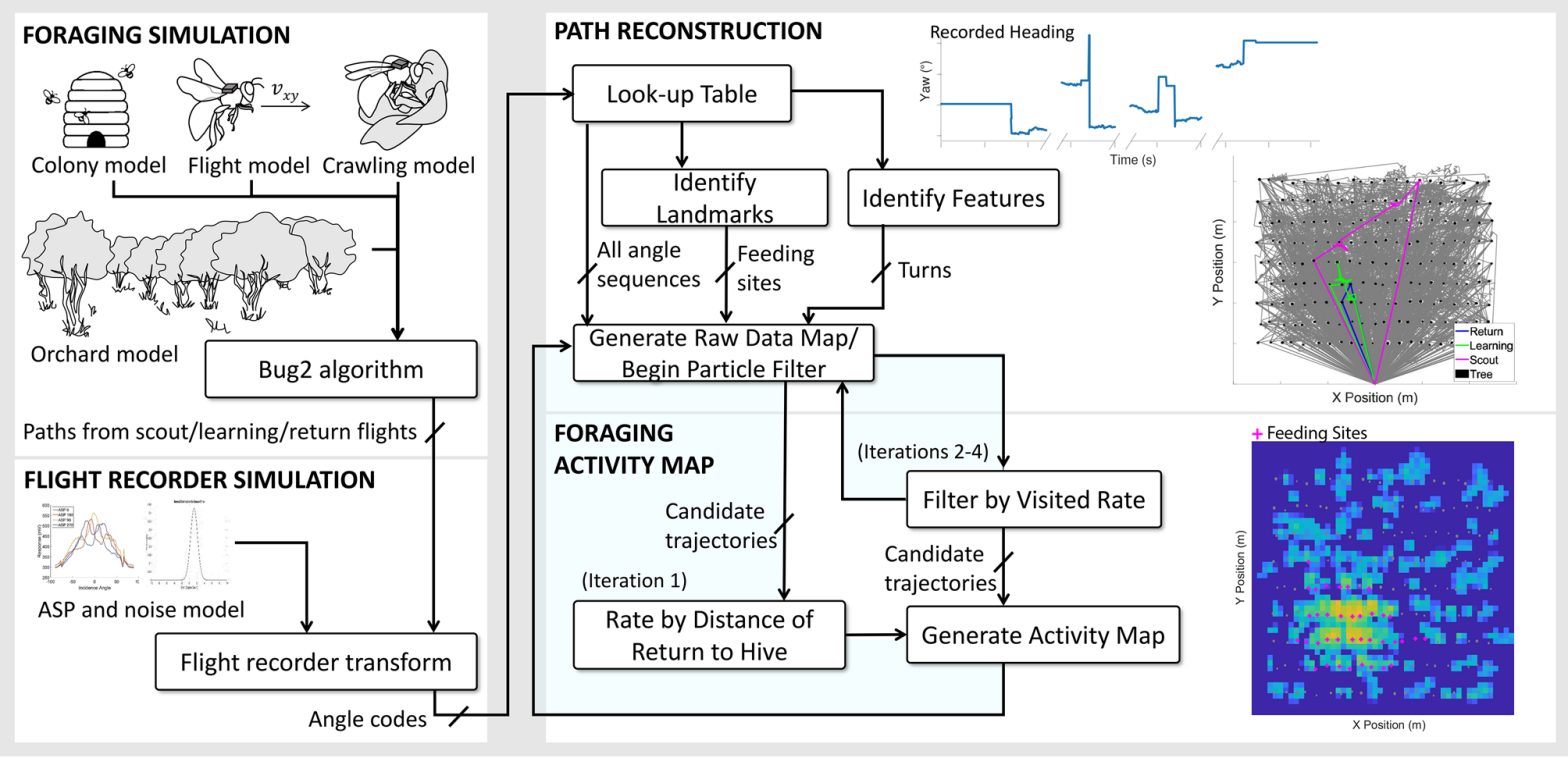
Flow chart combining the colony foraging simulation, simulated flight recorder, path reconstruction, and data processing to output the final foraging activity map. Recorded heading plot made in Python 3.7. Other plots were created in MATLAB^33^.

Path reconstruction inherently depends on the accuracy with which our sensor is able to describe the motion of an instrumented bee. Beyond limited angle resolution, errors related to the sampling rate accumulate when turns occur, at worst $v_{loaded} \Delta t$ = 1.6 m. To increase the accuracy of our foraging activity maps, we use models of sensor noise and flight speed, and leverage all recorded flights. The full workflow is shown in Fig. 6, where the foraging simulation portion generates the data we expect if our sensors are placed on actual bees, and the remaining flow is the data processing portion of our methodology.

We first identify feeding and turn features in our path data that stand out above the noise floor. Feeding features consist of crawling behavior in which the bee is moving at much lower speed compared to flying, but with rapidly varying yaw and AOI, inducing an elevated rate of change in measured AOI. A turn is marked by a significant change in yaw. Given the time of day and location, we can find the expected AOI on the orchard and use this to find the yaw from our lookup table. Our algorithm then indexes the sensor noise lookup table to find the related mean and standard deviation and uses this to estimate turns and feeding status. Our method marks a turn for a change greater than three standard deviations in yaw ($6.06^\circ$). A feeding site is marked if the detected AOI deviates more than three standard deviations ($3.72^\circ$) from the one expected, or if two consecutive AOI samples deviate more than 3 standard deviations, adjustable depending on the total flight time. The average detection accuracy of a turn is 99% with a standard deviation of 0.28% and average detection accuracy of a feeding site is 99% with a standard deviation of 0.29%.

We explore three methods to generate activity maps: from the raw data, we classify feeding features using the aforementioned statistical approach and produce maps based on accumulated path reconstructions; in “iteration 1” and “iteration 4” we take a particle filter approach to improve path localization based on knowledge of the hive and frequented feeding sites respectively. Particle filters are used to track a variable of interest over time by creating many representative particles, generating predictions according to dynamics and error models, and then updating them according to observation models^50^. In this case, each particle forms a candidate trajectory and predictions are based on speed, sensor readings, and the sensor error model. Assuming that we start without knowledge of the orchard, we build up an observation model by reconstructing and accumulating the raw flight paths (Fig. 6, raw data). To account for the fact that bees may turn at any point between sensor readings, we then upsample our sensor readings by a factor of 8, essentially producing 8 guesses for where the bee actually turned. Based on the artificially upsampled data and a random sample from the sensor noise distribution at a given AOI, we then generate the displacement in each path segment as follows:

6 $$\begin{aligned} \begin{bmatrix} \Delta x_{t} \\ \Delta y_{t} \end{bmatrix} = v \Delta t \begin{bmatrix} \cos {(\gamma _{t} + \gamma _{noise})} \\ \sin {(\gamma _{t} + \gamma _{noise})} \end{bmatrix} \end{aligned}$$

The final path is found as a cumulative sum of these displacements. We repeat this process to generate 5000 particles for each flight. The choice of 5000 is guided by our variable dimensions; the upsampling rate of 8 was the highest we could handle on a quadcore desktop computer with 16 GB RAM—to truly represent all potential turns we would need a number of particles equal to 8 to the power of the number of turns per flight. For reference, the average number of turns per flight is 13, thus the true representation in our sampling approach would require $8^{13}$ particles.

In iteration 1, we choose ten of these particles according to proximity to the hive upon return, and use the feeding features from these to form an initial foraging activity map, represented by a discrete grid with computed visit numbers. Note that in a typical localization approach a single particle, or average of several particles, is chosen as the final reconstruction. In our approach, we retain 10 different reconstructions for each individual path in order to better represent the distribution of points in a path due to sensor noise.

After this first pass, we repeat the process, but now filter particles by using the initial activity map as an observation model for the particle filter (Fig. 6, iteration 2–4). Specifically, we update particle probability at each detected feeding site by assigning the probability of the nearest grid cell in the map to the particle, and then sampling the particles by weight. At the end of the process, the ten particles with the highest probability product are used to construct an updated activity map.

## Results and discussion

To evaluate our methodology, we simulated (in MATLAB^33^) 50 independent orchard foraging scenarios with randomly assigned goals. We also studied the impact of having fewer instrumented honey bees, 1% and 3% respectively, by sampling 20% and 60% of all generated flights assuming no particular order for scout or regular foraging flights. This comparison also serves to evaluate a scenario where many of the tagged bees leave the hive and do not return, a possible result of high environmental stressors such as weather, predators, or pesticides.

**Figure 7:**
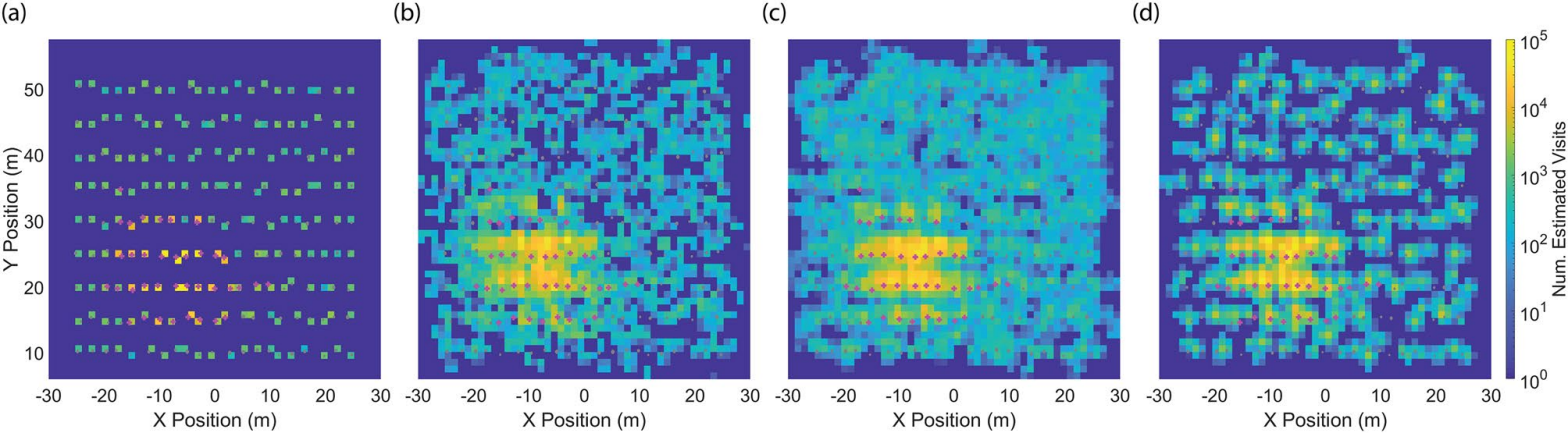
Example of generated activity maps. (**a**) the ground truth (**b**) estimated map from the raw data (**c**) estimated map after iteration 1 and (**d**) estimated map after iteration 4. Actual feeding sites are marked in magenta. Plots were created in MATLAB^33^.

Figure 7a shows an example outcome from 1750 flights, alongside estimated activity maps in Fig. 7b–d. We see that bees visit all trees in the orchard, but frequent actual goal sites and their neighboring trees more; this trend is also visible in maps generated from the raw data and iterations 1–4. Iteration 4 increases the computations by 4, but also shows a higher concentration of activity around the goal sites and significantly less activity in the empty areas between trees. This is a direct result of our re-iteration approach, which favors trajectories near high activity regions in the map.

**Figure 8:**
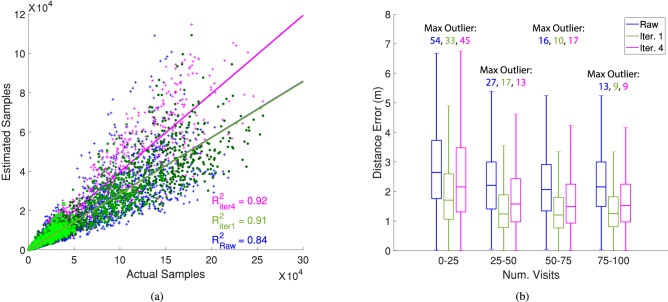
(**a**) The maximum number of estimated samples associated with a goal as a function of the number of actual samples for the raw data (blue), 1 (green), and 4 (magenta). Note that the linear fit for the raw data coincides with the fit for iteration 1. In iteration 1, data points are colored according to how many forager bees were instrumented: 5% (dark green), 3% (green), and 1% (bright green). (**b**) The distance of estimated visits from their true location as a function of number of visits. Plots were created in MATLAB^33^.

We next examine the accuracy of the raw data and iterations 1–4 by comparing the simulated flights with the reconstructed paths. We focus on two metrics: (1) the maximum number of estimated sensor samples compared to the true number of samples per visited tree in the map (Fig. 8a), and (2) the distance of the reconstructed feeding locations from their true location (Fig. 8b). In Fig. 8a, we show data from all iterations, but further break down iteration 1 by the number of tagged foragers in shades of green.

As expected, Fig. 8a shows a linear correlation between the number of estimated and true samples. Note that the large quantity of low count samples is due to the stark difference in activity by trees that are visited by scouts and actual goal locations that are visited on regular foraging flights. We see that the coefficient of determination is high (0.9) for iterations 1 and 4, and lower for the raw data (0.8) where measurement errors accumulate in every time step. We also note that the number of estimated and true samples have a higher correlation in iteration 4, although they are still off by an order of magnitude.

Figure 8b gives insights on the displacement error of the reconstructed feeding locations. We see that the mean and standard deviation, based on the median and interquartile distance, of the error is lowest for sites that have more than 25 visits. Simulations where only 1% or 3% of bees carry flight recorders still have many goals that exceed 25 visits and exhibit similar accuracy. Upon closer examination, trees with less than 25 visits correspond to ones that are most distant from initial goal sites or are visited only on scout flights, and because these visits typically occur late in the flights, they have also accumulated more error. We also note that the maximum outliers are inversely correlated with the number of visits as a result of less-visited goals occurring later in a given flight. We compute mean and standard deviation from the data presented in Fig. 8b and in comparing the raw data and iterations 1 and 4, find that the raw data always performs the worst with mean errors at more than 25 visits almost exceeding the distance between tree trunks ($\epsilon = 2.3 \pm 1.2\; \text{m}$). Iteration 1 performs the best ($\epsilon = 1.4 \pm 0.9 \; \text{m}$). Although iteration 4 improves the confidence in the cluster around a goal, it also has the detrimental effect of increasing the absolute error ($\epsilon = 1.8 \pm 1.2 \; \text{m}$). This happens because our re-iteration process does not associate feeding features with particular goals, but rather with any region of higher activity.

Our multi-iteration approach further enables different uses, depending on computational power and desired accuracy. The raw data yields information quickly with a sacrifice in accuracy. Iteration 1 requires longer processing, but offers improved accuracy for both single flights or end-of-day accumulated activity. Iteration 4 trades off accuracy and added computation with a better confidence in areas of activity, and could be combined with knowledge of specific bloom sites or with the information obtained from iteration 1 to better pinpoint activity.

Orchard pollination has strong implications for crop yield and management practices, yet monitoring these sporadic, brief, and spatially distributed events is very challenging. Instead of relying on high-end sensor and robotic technology to overcome this problem, we focused on applying Bayesian inference methods to data accumulated from large numbers of Angle-Sensitive Pixel arrays mounted on bee thoraxes, utilizing the highly cooperative nature of honey bee foragers who recruit other individuals from the colony to strategically exploit attractive food sources. Specifically, we presented system design considerations including (1) a mathematical model to help reason about chip memory versus sensor resolution given the strict limitations on overall chip area and weight given by the photovoltaic power output and honey bee size and payload; and (2) particle filter techniques to produce high level foraging activity maps given the coarse data returned from all sensors. To test our proposed system, we further implemented a comprehensive sensor and foraging simulator. We simulated a commercial colony with a small ($$<5\%$$) subset of instrumented foragers in a prototypical apple orchard, producing foraging maps that show high activity regions with 1.4–2.3 m accuracy, without assuming availability of standard tracking technology such as GPS, laser, or computer vision. Under the umbrella of digital agriculture, our approach is unique because it allows apple growers to measure honey bee pollination activity, which is correlated to fruit yield and profits, through instrumented colonies of honey bees^29^. Although we focused specifically on honey bees and orchards, similar potential may exist in other scenarios where swarms of agents (natural or robotic) already exhibit robust navigation in an unknown environment. A major strength of this approach is its cost effective and scalable nature: the data can be processed by a general-performance computer and we estimate that the flight recorder will cost $0.62 at research production level and likely significantly less at commercial scale.

In future work, we intend to test this technology with real colonies of honey bees, addressing the challenges of real flights and behaviors that are not captured in our current model such as changing flight speeds and diverse environmental conditions. This methodology is an important step towards the implementation of an inexpensive and scalable technology for analyzing and monitoring foraging activity in orchards, and represents an important thrust on leveraging natural organisms to complement current engineering practices in digital agriculture.

## Supplementary Information

Supplementary Information.
