# Supplementary material for: System design for inferring colony-level pollination activity through miniature bee-mounted sensors
Source: Sci Rep. 2021 Feb 19;11:4239. doi: 10.1038/s41598-021-82537-1 (PMC7895963; doi:10.1038/s41598-021-82537-1)
Supplement: Supplementary file 1 — Supplementary Information. [file 41598_2021_82537_MOESM1_ESM.pdf]

Supplementary Information: System Design for Inferring Colony-Level Pollination Activity through Miniature Bee-Mounted Sensors

Haron Abdel-Raziq[1,*], **Daniel Palmer**[1], **Phoebe Koenig**[1,2], **Alyosha Molnar**[1], **and Kirstin Petersen**[1]

[1]School of Electrical and Computer Engineering, Cornell University, Ithaca, NY 14850
[2]Department of Entymology, Cornell University, Ithaca, NY 14850
[*]corresponding author: hma49@cornell.edu

ABSTRACT

In digital agriculture, large-scale data acquisition and analysis can improve farm management by allowing growers to constantly monitor the state of a field. Deploying large autonomous robot teams to navigate and monitor cluttered environments, however, is difficult and costly. Here, we present methods that would allow us to leverage managed colonies of honey bees equipped with miniature flight recorders to monitor orchard pollination activity. Tracking honey bee flights can inform estimates of crop pollination, allowing growers to improve yield and resource allocation. Honey bees are adept at maneuvering complex environments and collectively pool information about nectar and pollen sources through thousands of daily flights. Additionally, colonies are present in orchards before and during bloom for many crops, as growers often rent hives to ensure successful pollination. We characterize existing Angle-Sensitive Pixels (ASPs) for use in flight recorders and calculate memory and resolution trade-offs. We further integrate ASP data into a colony foraging simulator and show how large numbers of flights refine system accuracy, using methods from robotic mapping literature. Our results indicate promising potential for such agricultural monitoring, where we leverage the superiority of social insects to sense the physical world, while providing data acquisition on par with explicitly engineered systems.

Appendix: Derivation of Trajectory Precision Equations

Section I: Derivation of Covariance Matrix of $\mathbf{p}_n$

The covariance matrix describing the precision of a position estimate $\mathbf{p}_n = \begin{bmatrix} x_n & y_n \end{bmatrix}^T$ given by our flight recorder can be derived from a basic model of honey bee motion. This motion model uses an assumed honey bee speed v and a sequence of heading measurements $\{\hat{\gamma}_0, \hat{\gamma}_1, ..., \hat{\gamma}_n\}$ to produce estimates of honey bee position over time. We present the covariance matrix associated with each sequential position estimate as a running sum of diagonalized matrices, a form that clearly shows the impact of each error source governing the performance of our flight recorder.

Several different error sources must be taken into account. First, heading measurements taken by the flight recorder will suffer from quantization error. To account for this error source, we denote the i_{th} heading measurement $\hat{\gamma}_i$, and we call the quantization error present in this measurement $\varepsilon_{\gamma,i}$. Second, the time between measurements will be subject to random variations stemming from noise processes in the integrated circuit at the core of the flight recorder. To capture this source of error, we denote the nominal timestep as $\hat{\Delta t}$ and the error in the i_{th} timestep as $\varepsilon_{\Delta t,i}$. These errors can be included in the honey bee motion model as follows:

$$\mathbf{p}_n = \sum_{i=0}^{n-1} v\mathbf{h}(\hat{\gamma}_i + \varepsilon_{\gamma,i})(\hat{\Delta t} + \varepsilon_{\Delta t,i}) \tag{A1}$$

where $\mathbf{h}$ is a unit vector pointing along the heading of the honey bee. By including these errors, one can derive the covariance matrix characterizing the position estimation precision achieved by the flight recorder.

The derivation begins with the definition of the covariance matrix for position estimate $\mathbf{p}_n = \begin{bmatrix} x_n & y_n \end{bmatrix}^T$:

$$\mathbf{\Sigma}_n = \mathrm{E}[(\mathbf{p}_n - \mathrm{E}[\mathbf{p}_n])(\mathbf{p}_n - \mathrm{E}[\mathbf{p}_n])^T] \tag{A2}$$

The motion model is nonlinear with respect to $\varepsilon_{\gamma,i}$, owing to the trignometric functions in $\mathbf{h}$. To simplify analysis, the motion model can first be linearized with respect to $\varepsilon_{\gamma,i}$:

$$\mathbf{p}_n = \sum_{i=0}^{n-1} v\mathbf{h}(\hat{\gamma}_i + \varepsilon_{\gamma,i})(\hat{\Delta t} + \varepsilon_{\Delta t,i}) \approx \sum_{i=0}^{n-1} [v\mathbf{h}(\hat{\gamma}_i)(\hat{\Delta t} + \varepsilon_{\Delta t,i}) + v\mathbf{h}^{\perp}(\hat{\gamma}_i)\varepsilon_{\gamma,i}(\hat{\Delta t} + \varepsilon_{\Delta t,i})] \tag{A3}$$

A convenient and illuminating form of the covariance matrix can be achieved by rewriting $\mathbf{p}_n$ in terms of the in terms of the 2 x 2 rotation matrix $R(\hat{\gamma}_i) = \begin{bmatrix} \cos(\hat{\gamma}_i) & -\sin(\hat{\gamma}_i) \\ \sin(\hat{\gamma}_i) & \cos(\hat{\gamma}_i) \end{bmatrix}$:

$$\mathbf{p}_n \approx \sum_{i=0}^{n-1} [v\mathbf{h}(\hat{\gamma}_i)(\hat{\Delta t} + \varepsilon_{\Delta t,i}) + v\mathbf{h}^{\perp}(\hat{\gamma}_i)\varepsilon_{\gamma,i}(\hat{\Delta t} + \varepsilon_{\Delta t,i})] \tag{A4}$$

$$= \sum_i [v\left(R(\hat{\gamma}_i)\begin{bmatrix}1\\0\end{bmatrix}\right)(\hat{\Delta t} + \varepsilon_{\Delta t,i}) + v\left(R(\hat{\gamma}_i)\begin{bmatrix}0\\ \varepsilon_{\gamma,i}\end{bmatrix}\right)(\hat{\Delta t} + \varepsilon_{\Delta t,i})] \tag{A5}$$

$$= \sum_i v\left(R(\hat{\gamma}_i)\begin{bmatrix}1\\ \varepsilon_{\gamma,i}\end{bmatrix}\right)(\hat{\Delta t} + \varepsilon_{\Delta t,i}) \tag{A6}$$

The expected value of $\mathbf{p}_n$ can then be written as

$$\mathrm{E}[\mathbf{p}_n] = \mathrm{E}\left[\sum_i v\left(R(\hat{\gamma}_i)\begin{bmatrix}1\\ \varepsilon_{\gamma,i}\end{bmatrix}\right)(\hat{\Delta t} + \varepsilon_{\Delta t,i})\right] = \sum_i vR(\hat{\gamma}_i)\begin{bmatrix}1\\0\end{bmatrix}\hat{\Delta t} \tag{A7}$$

since the heading discretization error $\varepsilon_{\gamma,i}$ and timing error $\varepsilon_{\Delta t,i}$ are both zero-mean. The difference between $\mathbf{p}_n$ and $\mathrm{E}[\mathbf{p}_n]$ is then:

$$\mathbf{p}_n - \mathrm{E}[\mathbf{p}_n] = \left(\sum_i vR(\hat{\gamma}_i)\begin{bmatrix}1\\ \varepsilon_{\gamma,i}\end{bmatrix}(\hat{\Delta t} + \varepsilon_{\Delta t,i})\right) - \left(\sum_i vR(\hat{\gamma}_i)\begin{bmatrix}1\\0\end{bmatrix}\hat{\Delta t}\right) = \sum_i vR(\hat{\gamma}_i)\begin{bmatrix}\varepsilon_{\Delta t,i}\\ \varepsilon_{\gamma,i}(\hat{\Delta t} + \varepsilon_{\Delta t,i})\end{bmatrix} \tag{A8}$$

The covariance matrix equation can then be evaluated:

$$\mathbf{\Sigma}_n = \mathrm{E}[(\mathbf{p}_n - \mathrm{E}[\mathbf{p}_n])(\mathbf{p}_n - \mathrm{E}[\mathbf{p}_n])^T] = \mathrm{E}\left[\left(\sum_i vR(\hat{\gamma}_i)\begin{bmatrix}\varepsilon_{\Delta t,i}\\ \varepsilon_{\gamma,i}(\hat{\Delta t} + \varepsilon_{\Delta t,i})\end{bmatrix}\right)\left(\sum_i vR(\hat{\gamma}_i)\begin{bmatrix}\varepsilon_{\Delta t,i}\\ \varepsilon_{\gamma,i}(\hat{\Delta t} + \varepsilon_{\Delta t,i})\end{bmatrix}\right)^T\right] \tag{A9}$$

This product-of-sums can be rewritten as a sum-of-products:

$$\mathbf{\Sigma}_n = \mathrm{E}\left[\sum_i\sum_j v^2R(\hat{\gamma}_i)\begin{bmatrix}\varepsilon_{\Delta t,i}\\ \varepsilon_{\gamma,i}(\hat{\Delta t} + \varepsilon_{\Delta t,i})\end{bmatrix}\begin{bmatrix}\varepsilon_{\Delta t,j} & \varepsilon_{\gamma,j}(\hat{\Delta t} + \varepsilon_{\Delta t,j})\end{bmatrix}R^T(\hat{\gamma}_j)\right] \tag{A10}$$

$$= \mathrm{E}\left[\sum_i\sum_j v^2R(\hat{\gamma}_i)\begin{bmatrix}\varepsilon_{\Delta t,i}\varepsilon_{\Delta t,j} & \varepsilon_{\gamma,j}\varepsilon_{\Delta t,i}(\hat{\Delta t} + \varepsilon_{\Delta t,j})\\ \varepsilon_{\gamma,i}\varepsilon_{\Delta t,j}(\hat{\Delta t} + \varepsilon_{\Delta t,i}) & \varepsilon_{\gamma,i}\varepsilon_{\gamma,j}(\hat{\Delta t} + \varepsilon_{\Delta t,i})(\hat{\Delta t} + \varepsilon_{\Delta t,j})\end{bmatrix}R^T(\hat{\gamma}_j)\right] \tag{A11}$$

Assuming independence of $\varepsilon_{\gamma,i}$ and $\varepsilon_{\Delta t,i}$, the sum can be re written as :

$$\mathbf{\Sigma}_n = \mathrm{E}\left[\sum_i\sum_j v^2R(\hat{\gamma}_i)\begin{bmatrix}\varepsilon_{\Delta t,i}\varepsilon_{\Delta t,j} & \varepsilon_{\gamma,j}\varepsilon_{\Delta t,i}(\hat{\Delta t} + \varepsilon_{\Delta t,j})\\ \varepsilon_{\gamma,i}\varepsilon_{\Delta t,j}(\hat{\Delta t} + \varepsilon_{\Delta t,i}) & \varepsilon_{\gamma,i}\varepsilon_{\gamma,j}(\hat{\Delta t} + \varepsilon_{\Delta t,i})(\hat{\Delta t} + \varepsilon_{\Delta t,j})\end{bmatrix}R^T(\hat{\gamma}_j)\right] \tag{A12}$$

This double summation can be separated into two parts:

$$\mathbf{\Sigma}_n = \mathrm{E}\left[\sum_{i,\, j=i} v^2R(\hat{\gamma}_i)\begin{bmatrix}\varepsilon_{\Delta t,i}\varepsilon_{\Delta t,j} & \varepsilon_{\gamma,j}\varepsilon_{\Delta t,i}(\hat{\Delta t} + \varepsilon_{\Delta t,j})\\ \varepsilon_{\gamma,i}\varepsilon_{\Delta t,j}(\hat{\Delta t} + \varepsilon_{\Delta t,i}) & \varepsilon_{\gamma,i}\varepsilon_{\gamma,j}(\hat{\Delta t} + \varepsilon_{\Delta t,i})(\hat{\Delta t} + \varepsilon_{\Delta t,j})\end{bmatrix}R^T(\hat{\gamma}_j)\right] \tag{A13}$$

$$+ \mathrm{E}\left[\sum_i\sum_{j,\, j\neq i} v^2R(\hat{\gamma}_i)\begin{bmatrix}\varepsilon_{\Delta t,i}\varepsilon_{\Delta t,j} & \varepsilon_{\gamma,j}\varepsilon_{\Delta t,i}(\hat{\Delta t} + \varepsilon_{\Delta t,j})\\ \varepsilon_{\gamma,i}\varepsilon_{\Delta t,j}(\hat{\Delta t} + \varepsilon_{\Delta t,i}) & \varepsilon_{\gamma,i}\varepsilon_{\gamma,j}(\hat{\Delta t} + \varepsilon_{\Delta t,i})(\hat{\Delta t} + \varepsilon_{\Delta t,j})\end{bmatrix}R^T(\hat{\gamma}_j)\right] \tag{A14}$$

The expectation operator can be applied to each element in the above sum of matrices. Since $\varepsilon_{\gamma,i}$ and $\varepsilon_{\Delta t,i}$ are zero-mean, the above equation can be rewritten as:

$$\mathbf{\Sigma}_n = \sum_i v^2 R(\hat{\gamma}_i) \begin{bmatrix} \mathrm{E}[\varepsilon_{\Delta t,i}^2] & 0 \\ 0 & \mathrm{E}[\varepsilon_{\gamma,i}^2(\hat{\Delta t}^2 + \varepsilon_{\Delta t,i}^2)] \end{bmatrix} R^T(\hat{\gamma}_j) \tag{A15}$$

$$+ \sum_i \sum_{j,\, j \neq i} v^2 R(\hat{\gamma}_i) \begin{bmatrix} \mathrm{E}\left[\varepsilon_{\Delta t,i}\varepsilon_{\Delta t,j}\right] & \mathrm{E}\left[\varepsilon_{\gamma,j}\varepsilon_{\Delta t,i}(\hat{\Delta t} + \varepsilon_{\Delta t,j})\right] \\ \mathrm{E}\left[\varepsilon_{\gamma,i}\varepsilon_{\Delta t,j}(\hat{\Delta t} + \varepsilon_{\Delta t,i})\right] & \mathrm{E}\left[\varepsilon_{\gamma,i}\varepsilon_{\gamma,j}(\hat{\Delta t} + \varepsilon_{\Delta t,i})(\hat{\Delta t} + \varepsilon_{\Delta t,j})\right] \end{bmatrix} R^T(\hat{\gamma}_j) \tag{A16}$$

Assuming no autocorrelation between successive measurements of $\varepsilon_{\gamma,i}$ and $\varepsilon_{\Delta t,i}$, respectively, the expectation operator in the second matrix above becomes separable:

$$\mathbf{\Sigma}_n = \sum_i v^2 R(\hat{\gamma}_i) \begin{bmatrix} \mathrm{E}[\varepsilon_{\Delta t,i}^2] & 0 \\ 0 & \mathrm{E}[\varepsilon_{\gamma,i}^2(\hat{\Delta t}^2 + \varepsilon_{\Delta t,i}^2)] \end{bmatrix} R^T(\hat{\gamma}_j) \tag{A17}$$

$$+ \sum_i \sum_{j,\, j \neq i} v^2 R(\hat{\gamma}_i) \begin{bmatrix} \mathrm{E}[\varepsilon_{\Delta t,i}]\mathrm{E}[\varepsilon_{\Delta t,j}] & \mathrm{E}[\varepsilon_{\gamma,j}]\mathrm{E}[\varepsilon_{\Delta t,i}]\mathrm{E}[(\hat{\Delta t} + \varepsilon_{\Delta t,j})] \\ \mathrm{E}[\varepsilon_{\gamma,i}]\mathrm{E}[\varepsilon_{\Delta t,j}]\mathrm{E}[(\hat{\Delta t} + \varepsilon_{\Delta t,i})] & \mathrm{E}[\varepsilon_{\gamma,i}]\mathrm{E}[\varepsilon_{\gamma,j}]\mathrm{E}[(\hat{\Delta t} + \varepsilon_{\Delta t,i})]\mathrm{E}[(\hat{\Delta t} + \varepsilon_{\Delta t,j})] \end{bmatrix} R^T(\hat{\gamma}_j) \tag{A18}$$

By the assumption of zero-mean error variables, all terms in the second matrix vanish, and we are left with the final result:

$$\mathbf{\Sigma}_n = \sum_i R(\hat{\gamma}_i) \begin{bmatrix} v^2\sigma_{\Delta t}^2 & 0 \\ 0 & v^2\sigma_\gamma^2(\hat{\Delta t}^2 + \sigma_{\Delta t}^2) \end{bmatrix} R^T(\hat{\gamma}_i) \tag{A19}$$

Thus, we obtain the covariance matrix at timestep n as a sum of diagonal matrices conjugated by rotation matrices that are evaluated on the sequence of measured headings.

Section II: Estimated error in straight-line trajectories bounds error for all trajectories of same length

Let $\{\sigma_{1,n}^*, \sigma_{2,n}^*\}$ denote the directed standard deviations computed at timestep n for a straight-line trajectory, and let $\{\sigma_{1,n}, \sigma_{2,n}\}$ denote the directed standard deviations computed at timestep n for an arbitrary trajectory. We aim to derive the following pair of results:

$$\min(\sigma_{1,n}^*, \sigma_{2,n}^*) \leq \min(\sigma_{1,n}, \sigma_{2,n}) \tag{A20}$$

$$\max(\sigma_{1,n}, \sigma_{2,n}) \leq \max(\sigma_{1,n}^*, \sigma_{2,n}^*) \tag{A21}$$

We begin by deriving $\sigma_{1,n}$ and $\sigma_{2,n}$ for an arbitrary sequence of measured headings

$$\mathbf{\Sigma}_n = \sum_{i=0}^{n-1} R(\hat{\gamma}_i)\,\mathbf{\Sigma}_0\,R^T(\hat{\gamma}_i) = \sum_{i=0}^{n-1} \begin{bmatrix} \cos(\hat{\gamma}_i) & -\sin(\hat{\gamma}_i) \\ \sin(\hat{\gamma}_i) & \cos(\hat{\gamma}_i) \end{bmatrix} \begin{bmatrix} \sigma_{11,0} & 0 \\ 0 & \sigma_{22,0} \end{bmatrix} \begin{bmatrix} \cos(\hat{\gamma}_i) & -\sin(\hat{\gamma}_i) \\ \sin(\hat{\gamma}_i) & \cos(\hat{\gamma}_i) \end{bmatrix}^T \tag{A22}$$

The product of the summand matrices can be computed:

$$\mathbf{\Sigma}_n = \begin{bmatrix} \sigma_{11,n} & \sigma_{12,n} \\ \sigma_{21,n} & \sigma_{22,n} \end{bmatrix} = \sum_{i=0}^{n-1} \begin{bmatrix} \sigma_{11,0}\cos^2(\hat{\gamma}_i) + \sigma_{22,0}\sin^2(\hat{\gamma}_i) & \sigma_{11,0}\cos(\hat{\gamma}_i)\sin(\hat{\gamma}_i) - \sigma_{22,0}\sin(\hat{\gamma}_i)\cos(\hat{\gamma}_i) \\ \sigma_{11,0}\sin(\hat{\gamma}_i)\cos(\hat{\gamma}_i) - \sigma_{22,0}\cos(\hat{\gamma}_i)\sin\hat{\gamma}_i) & \sigma_{11,0}\sin^2(\hat{\gamma}_i) + \sigma_{22,0}\cos^2(\hat{\gamma}_i) \end{bmatrix} \tag{A23}$$

Since the directed standard deviations are the square roots of the eigenvalues of $\mathbf{\Sigma}_n$, we wish to examine the eigenvalues of $\mathbf{\Sigma}_n$. These eigenvalues can be computed in the standard manner:

$$|\mathbf{\Sigma}_n - \lambda I| = \begin{vmatrix} \sigma_{11,n} - \lambda & \sigma_{12,n} \\ \sigma_{21,n} & \sigma_{22,n} - \lambda \end{vmatrix} = 0 \tag{A24}$$

Evaluating this determinant yields the characteristic equation for $\mathbf{\Sigma}_n$:

$$\lambda^2 - (\sigma_{11,n} + \sigma_{22,n})\lambda + (\sigma_{11,n}\sigma_{22,n} - \sigma_{12,n}\sigma_{21,n}) \tag{A25}$$

The eigenvalues of $\boldsymbol{\Sigma}_n$ are thus given by:

$$\lambda_{1,2} = \frac{(\sigma_{11,n} + \sigma_{22,n}) \pm \sqrt{(\sigma_{11,n} + \sigma_{22,n})^2 - 4(\sigma_{11,n}\sigma_{22,n} - \sigma_{12,n}\sigma_{21,n})}}{2} \tag{A26}$$

Since every covariance matrix is symmetric, we have $\sigma_{21,n} = \sigma_{12,n}$, and we can rewrite the above equation as

$$\lambda_{1,2} = \frac{(\sigma_{11,n} + \sigma_{22,n}) \pm \sqrt{(\sigma_{11,n} - \sigma_{22,n})^2 + (2\sigma_{12,n})^2}}{2} \tag{A27}$$

Observing each term in the above equation:

$$\sigma_{11,n} = \sum_i [\sigma_{11,0} \cos^2(\hat{\gamma}_i) + \sigma_{22,0} \sin^2(\hat{\gamma}_i)] \tag{A28}$$

$$\sigma_{12,n} = \sum_i [(\sigma_{11,0} - \sigma_{22,0}) \sin(\hat{\gamma}_i) \cos(\hat{\gamma}_i)] \tag{A29}$$

$$\sigma_{22,n} = \sum_i [\sigma_{11,0} \sin^2(\hat{\gamma}_i) + \sigma_{22,0} \cos^2(\hat{\gamma}_i)] \tag{A30}$$

We can evaluate the quadratic formula term-by-term:

Term I:

$$(\sigma_{11,n} + \sigma_{22,n}) = \sum_i [\sigma_{11,0} \cos^2(\hat{\gamma}_i) + \sigma_{22,0} \sin^2(\hat{\gamma}_i)] + \sum_i [\sigma_{11,0} \sin^2(\hat{\gamma}_i) + \sigma_{22,0} \cos^2(\hat{\gamma}_i)] \tag{A31}$$

$$= \sum_i [\sigma_{11,0}(\cos^2(\hat{\gamma}_i) + \sin^2(\hat{\gamma}_i)) + \sigma_{22,0}(\cos^2(\hat{\gamma}_i) + \sin^2(\hat{\gamma}_i))] \tag{A32}$$

$$= \sum_i [\sigma_{11,0} + \sigma_{22,0}] \tag{A33}$$

$$= n[\sigma_{11,0} + \sigma_{22,0}] \tag{A34}$$

Term II:

$$(\sigma_{11,n} - \sigma_{22,n}) = \sum_i [\sigma_{11,0} \cos^2(\hat{\gamma}_i) + \sigma_{22,0} \sin^2(\hat{\gamma}_i)] - \sum_i [\sigma_{11,0} \sin^2(\hat{\gamma}_i) + \sigma_{22,0} \cos^2(\hat{\gamma}_i)] \tag{A35}$$

$$= \sum_i \sigma_{11,0}[\cos^2(\hat{\gamma}_i) - \sin^2(\hat{\gamma}_i)] + \sum_i \sigma_{22,0}[\sin^2(\hat{\gamma}_i) - \cos^2(\hat{\gamma}_i)] \tag{A36}$$

$$= \sum_i [(\sigma_{11,0} - \sigma_{22,0})(\cos^2(\hat{\gamma}_i) - \sin^2(\hat{\gamma}_i))] \tag{A37}$$

$$= \sum_i [(\sigma_{11,0} - \sigma_{22,0}) \cos(2\hat{\gamma}_i)] \tag{A38}$$

$$= (\sigma_{11,0} - \sigma_{22,0}) \left(\sum_i \cos(2\hat{\gamma}_i) \right) \tag{A39}$$

Term III:

$$2\sigma_{12,n} = 2\sum_i (\sigma_{11,0} - \sigma_{22,0}) \sin(\hat{\gamma}_i) \cos(\hat{\gamma}_i) \tag{A40}$$

$$= \sum_i (\sigma_{11,0} - \sigma_{22,0}) 2 \sin(\hat{\gamma}_i) \cos(\hat{\gamma}_i) \tag{A41}$$

$$= \sum_i (\sigma_{11,0} - \sigma_{22,0})(\sin(2\hat{\gamma}_i)) \tag{A42}$$

$$= (\sigma_{11,0} - \sigma_{22,0}) \left(\sum_i \sin(2\hat{\gamma}_i) \right) \tag{A43}$$

The eigenvalue equation then becomes:

$$\lambda_{1,2} = \frac{n(\sigma_{11,0}+\sigma_{22,0}) \pm \sqrt{(\sigma_{11,0}-\sigma_{22,0})^2(\sum_i \cos(2\hat{\gamma}_i))^2 + (\sigma_{11,0}-\sigma_{22,0})^2(\sum_i \sin(2\hat{\gamma}_i))^2}}{2} \tag{A44}$$

$$= \frac{n(\sigma_{11,0}+\sigma_{22,0}) \pm (\sigma_{11,0}-\sigma_{22,0})\sqrt{(\sum_i \cos(2\hat{\gamma}_i))^2 + (\sum_i \sin(2\hat{\gamma}_i))^2}}{2} \tag{A45}$$

The eigenvalues $\lambda_{1,2}$ achieve their extreme values when the term $((\sigma_{11,0}-\sigma_{22,0})\sqrt{(\sum_i \cos(2\hat{\gamma}_i))^2 + (\sum_i \sin(2\hat{\gamma}_i))^2})$ is maximized with respect to $\hat{\gamma}_i$. The $\hat{\gamma}_i$-dependent terms within this expression can be rewritten as:

$$(\sum_i \cos(2\hat{\gamma}_i))^2 + (\sum_i \sin(2\hat{\gamma}_i))^2 = \sum_i \sum_j \cos(2\hat{\gamma}_i)\cos(2\hat{\gamma}_j) + \sum_i \sum_j \sin(2\hat{\gamma}_i)\sin(2\hat{\gamma}_j) \tag{A46}$$

$$= \sum_i \sum_j [\cos(2\hat{\gamma}_i)\cos(2\hat{\gamma}_j) + \sin(2\hat{\gamma}_i)\sin(2\hat{\gamma}_j)] \tag{A47}$$

$$= \sum_i \sum_j [\frac{1}{2}[\cos(2(\hat{\gamma}_i - \hat{\gamma}_j)) + \cos(2(\hat{\gamma}_i + \hat{\gamma}_j))] + \frac{1}{2}[\cos(2(\hat{\gamma}_i - \hat{\gamma}_j)) - \cos(2(\hat{\gamma}_i + \hat{\gamma}_i))]] \tag{A48}$$

$$= \sum_i \sum_j [\cos(2(\hat{\gamma}_i - \hat{\gamma}_j))] \tag{A49}$$

This sum maximizes if $\hat{\gamma}_i = \hat{\gamma}_j = \hat{\gamma}_0 \; \forall \{i,j\}$, thus demonstrating that $\lambda_{1,2}$ co-extremize for straight-line trajectories. Since the directed standard deviations are the square roots of the eigenvalues of the covariance matrix, we have therefore shown that straight-line directed standard deviations $\sigma^*_{1,n}$ and $\sigma^*_{2,n}$ provide upper and lower bounds on the directed standard deviations for any sequence of $\hat{\gamma}_i$.

Appendix: Orchard Modeling

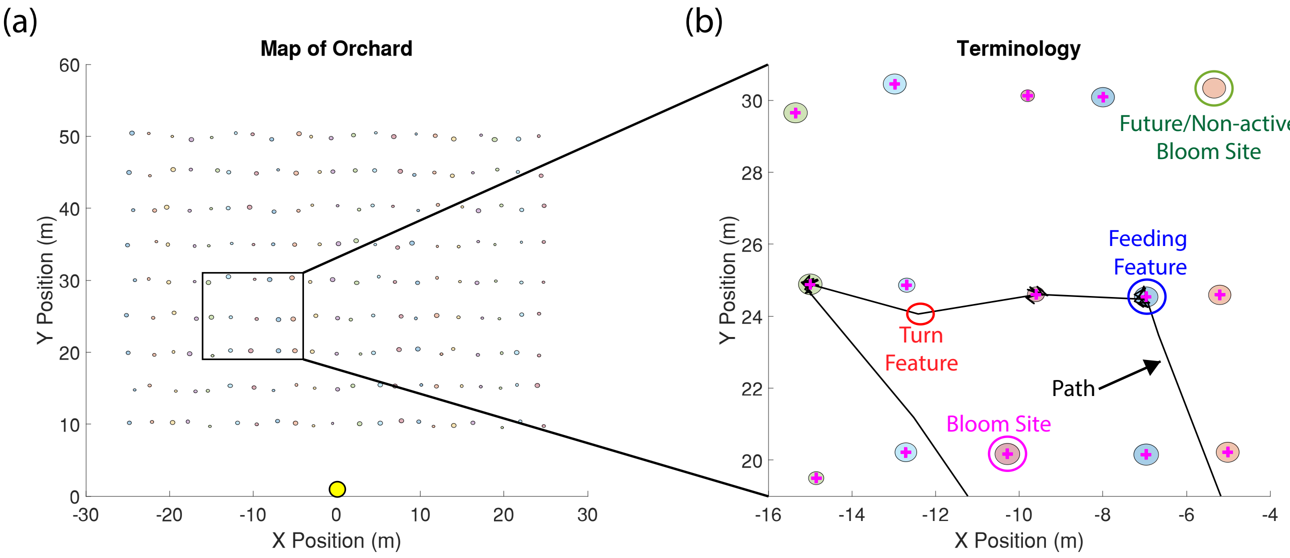

Figure S1. Overview of orchard model. (a) Model orchard generated in MATLAB[1]. (b) Zoomed in of region from (a) illustrating model parameters.

In the main article, we introduced and discussed an orchard model created in MATLAB[1] for simulating honey bee flights. This section will present further details of our model as shown in Fig. S1. The circles in (Fig. S1-a) represent an aerial view of trees in our MATLAB[1] simulation. As mentioned in the main article, the radii are assigned randomly based on reported tree parameters[2], with vertical and horizontal spacing also assigned randomly based on reported numbers. The hive is the yellow circle situated at $(0,0)$. In the main text, we discuss the terms denoting various parts of our model, but in Fig. S1-b, we outline this in an image for the reader. We zoom in on part of the map and overlay a honey bee path, with text describing what each

item on the map is in terms of our model. We hope this section will further aid a reader in designing a similar simulation setup to that described in this work.

Appendix: Hive Setup

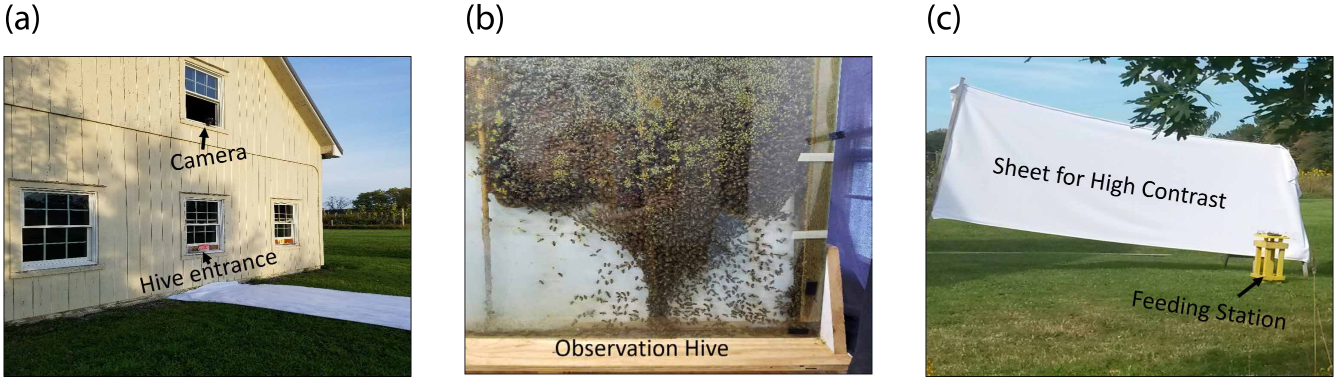

Figure S2. Setup for capturing honey bee flight trajectory data. (a) Hive entrance and camera position. (b) Observation hive inside of barn in (a). (c) Setup at feeder.

The section Honey Bee Motion Model in the main article describes our own investigation into honey bee trajectories. In this section, we present our setup for obtaining that data through Fig. S2. Honey bee facilities at Cornell University, seen in Fig. S2-a, were used for capturing all honey bee flight data. This consisted of a barn with several observation hives, with one selected for recording. A close-up of one observation hive can be seen in Fig. S2-b. Data for behavior near the hive was collected near the barn in Fig. S2-a while data for feeding behavior was collected near the site in Fig. S2-c. Flight data from hive to a feeding source was collected at several points in-between Fig. S2(a & c). Video data was then processed by MATLAB's[1] computer vision toolbox and computations performed to find the data reported in the main article.

Appendix: Reduced Flight Number Study

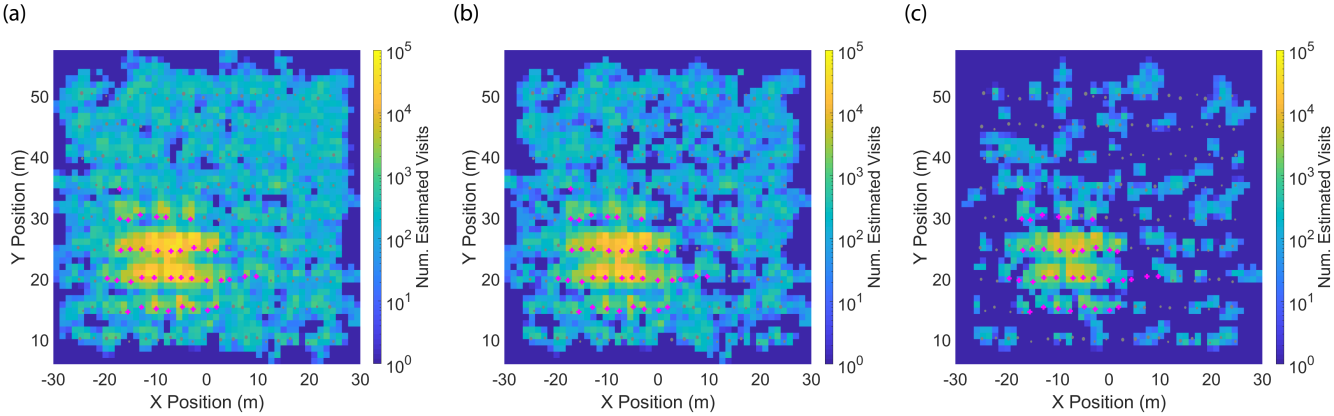

Figure S3. Example of generated activity maps assuming differing forager counts for Iteration 1, with full (1750 flights) in a) and the estimated maps after 1050 flights (b), and 350 flights (c). Actual feeding sites are marked in magenta. Plots were created in MATLAB[1].

Since tagging honey bees is often performed by hand in biological studies/settings, we examined the maps produced assuming fewer tagged foragers. This is important in the case a grower does not have the resources necessary to tag larger counts of bees or if tagged bees are lost due to predation, senescence, stress, and other factors. We studied the impact of having fewer instrumented honey bees, 1% and 3% respectively, by sampling 20% and 60% of all generated flights assuming no particular order for scout or regular foraging flights. In Fig. S3, for iteration 1, we see a comparison between full tagged flights (Fig. S3-a), which assumes 5% of all foragers are tagged, 3% of foragers tagged in Fig. S3-b, and 1% of foragers in Fig. S3-c.

The most striking feature of all three maps is the high activity region. Despite reduced forager counts, we can see similar regions of high activity. We further verify the usefulness of the information contained in these maps by examining the distance

error in Fig. S4 as performed in the main article. We note that there is little change in this distance metric whether 1%, 3%, or 5% of foragers are tagged. Consequently, the flight data returned by any of these forager counts, when mapped, yields high activity regions within a mean distance of 1.4*m* of the true location of the feeding sites. This shows our approach can be useful even if there is reduced forager tagging capability.

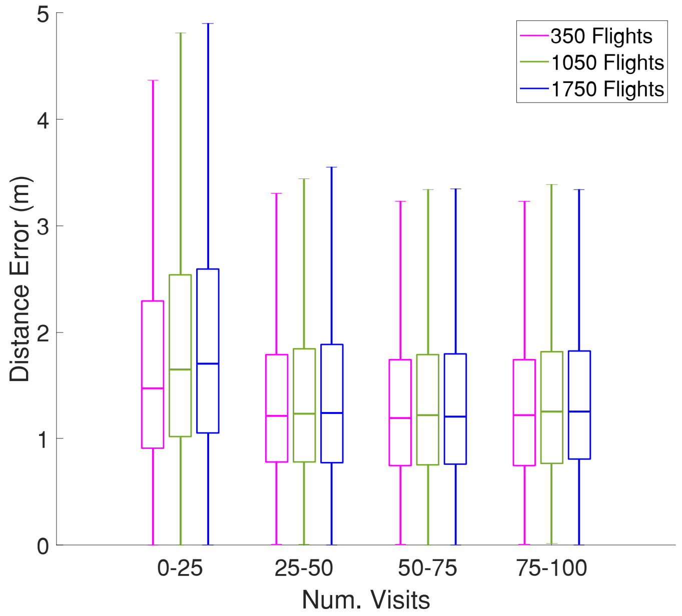

Figure S4. The distance of estimated visits from their true location for iteration 1 as a function of number of visits for 1750 flights (blue), 1050 flights (green), 350 flights (magenta). Plots were created in MATLAB[1].

References

1. MathWorks. MATLAB R2019a. https://www.mathworks.com/products/matlab.html (2019).
2. University of Vermont. Tree fruit: Practical guide for organic apple production. http://www.uvm.edu/~fruit/?Page=treefruit/tf_organicbasics/PracticalGuideOrganic/PracticalGuideOrganicHorticulture.html&SM=tf_submenu.html (2019).
